# School Bonds and the Onset of Substance Use among Korean Youth: An Examination of Social Control Theory

**DOI:** 10.3390/ijerph120302923

**Published:** 2015-03-09

**Authors:** Yoonsun Han, Heejoo Kim, Julie Ma

**Affiliations:** 1 Department of Child Psychology and Education, Sungkyunkwan University, 25-2 Sungkyunkwan-ro, Jongno-gu, Seoul, 110-745, Korea; E-Mail: kimhee@skku.edu; 2 School of Social Work, Michigan State University, 254 Baker Hall, East Lansing, MI 48824, USA; E-Mail: maju@msu.edu

**Keywords:** initiation of alcohol drinking, initiation of cigarette smoking, school bonds, social control theory, Korean adolescents

## Abstract

This study examined the association between school bonds and the onset of substance use among adolescents in South Korea. Based on Hirschi’s social control theory, this study tested the roles of teacher attachment, educational aspiration, extracurricular activities, and rule internalization—four elements of social bonds within the school setting—in delayed initiation of alcohol drinking and cigarette smoking. Discrete-time logistic regression was used to analyze five waves of the Korea Youth Panel Survey (*N* = 3449 at baseline), a nationally representative sample of Korean youth. Stronger teacher attachment, higher educational aspiration, and higher rule internalization were correlated with delayed onset of alcohol drinking and cigarette smoking. On the other hand, participation in school extracurricular activities was positively associated with the onset of alcohol drinking, but not statistically significantly linked with the onset of cigarette smoking. These findings suggest that early prevention strategies for youth substance use should specifically target school-related factors that represent social bonds developed among youth.

## 1. Introduction

This study examined the role of school factors based on the social control theoretical perspective [1] in seeking to understand patterns of first-time substance use behaviors among South Korean (Korea hereafter) adolescents. This topic is particularly timely given the high prevalence of underage drinking and smoking among adolescent populations in Korea. Despite legislative efforts through the Juvenile Protection Act to prevent adolescent substance use, the proportion of youth that have lifetime experience with alcohol and cigarette consumption has reached 47% and 25%, respectively [2]. Additionally, a national study of Korean youth reported that 14% of high school males had engaged in “severe” levels of alcohol consumption (that is, on average more than five shots of soju per episode; soju is a popular alcoholic beverage with about 20% alcohol by volume) in the past 30 days, and 10% of high school males had smoked every day for the past 30 days [3].

Underage alcohol drinking and cigarette smoking are of particular public health concern, as extant research has highlighted the associations between substance use in adolescence and a range of subsequent adverse outcomes including involvement in bullying [4], elevated depressive symptoms [5], risky sexual behaviors [6], suicidal ideation and attempts [7,8]. Furthermore, substance use behaviors that are formed during an early developmental period are likely to extend into adulthood [9] and in turn are at higher risk for progressing to illicit drug use in adulthood [10]. This continuum between youth and adult substance use is especially salient among adults who are abusive and dependent substance users [11,12]. Currently, Korea stands as one of the highest in terms of male adult smokers (more than 40% smoke daily) among all Organization for Economic Co-operation and Development countries [13,14]. Considering the high rate of substance use among adult populations, and the fact that adult risk behaviors are often formed at a young age, one may naturally point to the importance of providing a research basis that can guide prevention and intervention during adolescence.

The school domain may be central for understanding patterns of early substance use among youth populations. Further, schools are considered an effective and malleable target for prevention of and intervention in youth risk behaviors [15]. This is particularly so, as the school emerges as a critical ecological environment when children transition into adolescents [16]. Not only do adolescents spend a greater proportion of their time at school [17], but they also develop core relationships and bonds with teachers and peers within the school setting [18]. Furthermore, at school, adolescents have the opportunity to form future aspirations and internalize value systems that prevail in the larger society, beyond the family environment. Numerous theoretical and empirical works have documented that students with poorer connections to school showed more depressive symptoms, higher rates of substance use, and lower rates of school completion [15,19,20,21,22]. On the other hand, active student participation and positive relationships with others in school were linked with a reduction in negative outcomes, such as drug use [23].

Thus, given the crucial role of the school environment in youth outcomes, this study attempts to identify whether school-related factors are associated with first-time engagement in substance use behaviors among Korean adolescents. Although both prevalence and onset address key areas of youth substance use patterns and its correlates, they focus on markedly different aspects of risk behavior. Prevalence measures the extent to which youth engage in substances during a given period of time, whereas onset pinpoints to the exact timing or occurrence of an event. These two indicators do not necessarily occur during the same period. For example, although prevalence of cigarettes and alcohol use among Korean youth is highest during high school, first exposure is most common during second year of middle school [2]. Research suggests that understanding correlates of first-time onset of alcohol or cigarette use can be particularly resourceful for identifying ways to guide preventive intervention strategies [24].

In the following sections, we first present a theoretical framework based on social control theory followed by empirical evidence concerning the ways in which teacher attachment, educational aspiration, extracurricular activities, and rule internalization may predict youth substance use behaviors. Discrete-time logistic regression is used to examine whether and to what extent these various school domains are related to first-time initiation of alcohol drinking and cigarette smoking. Finally, the implications of the findings are discussed.

### 1.1. Social Control Theory

One of the leading theoretical lenses for understanding youth risk behaviors is Hirschi’s social control theory [25]. According to Hirschi, control theorists are primarily concerned with why individuals do *not* engage in risk behavior. Hirschi asserts that robust bonds that youth have to society constrict individual impulses toward delinquent actions. In other words, the decision to conform or deviate depends on the extent to which individuals have established bonds to conventional society. When these social bonds are weak or absent, individuals are more likely to participate in risk behavior.

Hirschi specified four major elements that comprise an individual’s bonds to society—attachment, commitment, involvement, and belief. *Attachment* refers to bonds with significant others, such as parents and teachers. Hirschi claims that “[i]f a person does not care about the wishes and expectations of other people … then he is to that extent not bound by the norms. He is free to deviate” ([1], p. 18). In this sense, youth who develop strong interpersonal connections with significant adult figures, such as school teachers, may refrain from delinquent behavior, as it is considered a threat that jeopardizes their well-established relationships. In support of this idea, previous research has found that positive youth-teacher attachment in the school setting is a protective factor in various youth misconduct, such as smoking, drug use, aggression, and theft [22,26]. For example, a study on early adolescents in an urban school district in the United States found that poor relationships with teachers were linked to poorer scores in social and emotional adjustment, which are connected to issues such as depression and delinquency [27].

*Commitment* is described as the time and energy that individuals invest in activities related to education, employment, or reputation. When determining one’s course of action, the individual “must consider the costs of deviant behavior, the risk he runs of losing the investment he has made in conventional behavior” ( [1], p. 20). In other words, individuals, as rational actors, actively weigh the costs and benefits associated with deviating from social norms. Thus, highly committed youth who value the norms and goals of conventional institutions, such as the school, may rationally decide to not engage in risk behaviors. According to Hirschi, educational or occupational aspirations serve as a source of motivation that impedes risky actions that would threaten valuable investments made by youth. Empirical research has confirmed that youth who demonstrate strong commitment to social values are less likely to act against the rules of society [20,28].

*Involvement* refers to the time and energy an individual spends participating in conventional activities, especially those related to school, to satisfy their recreational interests, which in turn reduces delinquency. Youth with high levels of involvement do not engage in delinquency as they are “simply too busy” participating in conventional activities ([1], p. 22). Involvement in conventional activities reduces one’s time to engage in risk behavior. Additionally, involvement enables youth to build ties with adult figures at the school, with whom youth can develop bonds and mutual trust and receive support, which in turn inhibits risk behaviors [29]. Some examples of involvement in conventional activities that have been empirically tested to be linked with attenuated levels of delinquency include participation in extracurricular activities, athletics [30,31], recreation and performing arts [32], religious activities [20], and community service [33].

The last element of social bond is *belief*, which is the extent to which youth accept rules and values that widely exist in society [1]. Control theorists claim that individuals vary in the extent to which they believe in the “moral validity of social rules” ([1], p. 26). Individuals with weak belief in social rules and values are more likely to violate them, whereas those who demonstrate strong internalization of societal beliefs abide by them. In the empirical literature base, a broad array of indicators of youth acceptance, abidance, and internalization of moral societal values has been used. Some of these examples include respect for the local police and law [1], attitude toward risk behavior [34], evaluation of school rules [35], and belief in the legal system [36]. In accordance with Hirschi’s idea, prior literature has found that youth with strong belief in conventional regulatory systems and values are less likely to be associated with negative outcomes, such as theft and violence [36] and school misbehavior [35]. Also, in the school setting, previous research indicates that strong belief in or adaptation to school rules or regulations is linked with reduced use of substances [37,38].

### 1.2. Contribution and Hypothesis

In sum, existent literature suggests that social control theory and its specific elements of social bonds provide an effective lens for understanding youth involvement in risk behaviors, including alcohol and cigarette use. Nonetheless, there are several limitations to the existing literature base, which the current study attempts to address. First, the vast majority of prior research has examined the link between social bond elements and the prevalence of risk behaviors, but not their onset. Of the few studies that have examined the correlates of the onset of substance use [24,39,40], only a limited number explain youth risk behaviors from a social control perspective. Most studies that apply social control theory to explain youth risk behaviors have only tested a subset of the four social bond elements—e.g., the relation of attachment to youth outcomes [41], predicting impact of social and school connectedness on later substance use [19], or comparison of the impacts of social belonging and teacher support on health-risk behaviors [42]—which limits our ability to compare the effects of each element [21]. Finally, with the exception of a few [34,43,44,45], the vast majority of empirical studies that apply social control theory to comprehend substance use behaviors among youth populations have been conducted in western countries [46,47], which limits the applicability of the theory to a broader global population.

This study addresses the above-mentioned limitations in the literature by investigating the associations between social control elements (attachment, commitment, involvement, and belief) and the onset of substance use behaviors using event history analysis based on longitudinal data from a Korean youth sample. In accordance with the predictions of social control theory, we hypothesize that the four elements of social bonds within the school domain would predict a delayed onset of alcohol and cigarette use among adolescents. Specifically, based on theoretical and empirical evidence, we carefully selected teacher attachment, educational aspiration, school extracurricular activities, and rule internalization to respectively represent the attachment, commitment, involvement, and belief elements. To the best of our knowledge, no other study has examined this topic using a nationally representative sample of Korean youth.

## 2. Materials and Methods

### 2.1. Data

Analyses were based on data from the nationally representative Korea Youth Panel Survey (KYPS) collected by the National Youth Policy Institute in Korea. Using stratified multi-stage cluster sampling, KYPS originally sampled 3449 second-year middle school students and their parents in 2003 (wave 1) and followed these individuals for six consecutive years until 2008 (wave 6). The follow-up rate between wave 1 and wave 6 was 71.3%. Although the original study gathered information across 6 waves, the present study used information from waves 1–5, as this is the period during which youth were in middle school and high school (8th–12th grade).

This prospective panel survey contained information concerning each youth’s individual, family, school, and neighborhood characteristics. A special feature of the KYPS is that in wave 1, youth were asked whether they had experienced alcohol drinking or cigarette smoking prior to the time of the survey. Youth who responded “yes” to this question were subsequently asked to report the age that they engaged in either alcohol drinking or cigarette smoking for the first time. This retrospective information on substance use initiation was used to reconstruct two person-period datasets suitable for event history analysis [48] to examine onset of first-time alcohol drinking and first-time cigarette smoking, respectively (details are mentioned in the Analysis Strategy section).

### 2.2. Measures

#### 2.2.1. Dependent Variables

Two substance use outcomes reported by the youth were examined in the study. The first dependent variable, *alcohol drinking*, was measured as “1” if the youth reported to have consumed alcohol in the past year for the first time in their life and “0” otherwise. The second dependent variable, *cigarette smoking*, was measured as “1” if the youth reported to have smoked cigarettes in the past year for the first time in their life and “0” if they had not. 

#### 2.2.2. Independent Variables

In accordance with social control theory [1], the current study investigated the role of four measures representing four different elements of social bonds, including attachment, commitment, involvement, and belief. All of the variables representing social bonds were reported by the youth.

The first measure, *teacher attachment*, represented the attachment element of social bonds within the school setting and reflected the degree to which youth feel connected to their teachers [1]. Three teacher-related items (e.g., “I can talk about all my troubles and worries to my teachers without reservation,” “Teachers treat me with love and affection,” “I hope to become a person just like my teacher”) were extracted from the original school-adjustment scale. Each item was answered on a five-point Likert scale (1 = very untrue; 2 = somewhat untrue; 3 = neither true nor untrue; 4 = somewhat true; 5 = very true). Response to the three items were equally weighted and combined into a single index. High scores indicate higher levels of positive teacher attachment. The internal consistency of the three items was 0.75.

The second measure, *educational aspiration*, is an indicator of the commitment element [1]. Youth were asked a single question: “How much education would you like to receive?” Their response to the question was measured in 5 levels (1 = junior high school; 2 = high school; 3 = junior college; 4 = college or university; 5 = Master’s or Ph.D. degree). For parsimony, this ordinal variable was entered as a single continuous measure in the model.

Involvement in school *extracurricular activities* can represent one type of conventional activity that strengthens adolescent bonds with society [15]. Youth were asked whether they participated in official school extracurricular activities. Involvement was measured as a binary variable (0 = no; 1 = yes).

The final social bond element, *belief*, reflects the degree to which youth agree with and internalize common rules and values in the society [1]. Belief was assessed using the following statement: “I find it difficult to follow school rules and regulations.” This five-point Likert scale was reverse-coded, so a higher score indicated higher levels of *rule internalization* (1 = very true; 2 = somewhat true; 3 = neither true nor untrue; 4 = somewhat untrue; 5 = very untrue).

#### 2.2.3. Control Variables

In consideration of prior work that addressed the importance of considering demographics [21], family socioeconomic status [49,50], and family structure [51] in models for understanding youth substance-use behavior, the following measures were included as statistical controls. *Average monthly family income* was measured in Korean currency (unit is 1,000,000 Korean won). *Parent’s education*, was measured as the highest level of completed education by either the mother or father (1 = no schooling; 2 = elementary school; 3 = middle school; 4 = high school; 5 = junior college (2–3 years); 6 = college or university (4 years); 7 = master’s degree; 8 = doctoral degree). For parsimony, this ordinal variable was entered as a single continuous measure in the analytic model, instead of seven dummy variables. *Family structure* was represented by two dummy variables (living with both parents, father only, mother only; living with both parents as the reference group). *Male* youth were coded as “1” and female as “0.” *Age*, an indicator of duration, was measured in number of years and was represented by four dummy variables (age 15 is reference group) in the model. Youth gender and age measures were obtained via youth self-report. All other control variables were reported by the primary caregiver of the youth participants.

### 2.3. Analysis Strategy

A summary of the descriptive characteristics of the sample youth at baseline (Table 1), Pearson correlation coefficients among the school bond variables (Table 2), and frequency and cumulative percentage of age of first initiation of substance use (Table 3) were presented. Next, we estimated a discrete-time hazard model with logistic regression to identify the predictors of the first occurrence of alcohol and cigarette use (Table 4). The analytic model is specified below [52]:

$$\ln\left(\frac{h_{it}}{1-h_{it}}\right)=\sum_{t=15}^{19}\alpha_{t}\left(Age_{it}\right)+\beta X_{it}$$

In the above model, *h_it_* is the hazard of first-time substance use for youth *i* at age *t*, *Age_it_* is a dummy variable for youth *i* at age *t*, and *X_it_* is a vector of time-variant school-bond elements (e.g., teacher attachment, educational aspiration, extracurricular activity, and rule internalization) and covariates (e.g., male, monthly family income, parental education, family structure) for youth *i* at age *t*. Estimated coefficients are represented by α_t_ for *Age_it_* (identifies the shape of the baseline hazard curve) and β for time-variant variables (shows the relationship between school bond elements and onset of substance use, while controlling for demographic and socioeconomic factors). 

Discrete time event history analysis is ideal for analyses in which the timing of an event, rather than its occurrence, is of importance to the researcher [53]. This method is proven to be particularly useful when examining substance use outcomes, since age is reported as a time interval rather than an exact age [52]. Unlike continuous time models, there are few possible time intervals with many individuals sharing the same time of event [54]. Probability sampling weights were used for the logistic regression models. Results were reported as logistic coefficients. Two person-period data sets were constructed to analyze the onset of alcohol drinking and the onset of cigarette consumption, respectively. For each dataset, there was one observation for each year from age 15 to age 19, until the age youth reported of their first drinking in the alcohol model and smoking experience in the cigarette model. Once the individual experienced first-time substance use, the individual was no longer “at risk” of first-time substance use, and thus was no longer observed. Thus, youth who had never used substances by the end of the survey (a.k.a., right censored) contained all five person-period observations. Additionally, youth respondents who had already experienced first-time use of alcohol or cigarettes prior to the first wave of the panel study were excluded from the survival analysis (a.k.a., left-censored). This is because retrospective information, other than age of first-time substance use, was not available for other covariates in the model (e.g., teacher attachment, educational aspiration, school extracurricular activities, and rule internalization) in the years prior to the baseline survey. This resulted in a sample size of 2870 youth (9242 person-years when including repeated observations) for the alcohol-drinking model and 3210 (12,972 person-years when including repeated observations) youth for the cigarette-smoking model.

## 3. Results

### 3.1. Descriptive Statistics

At baseline, all respondents were in 8th grade. There were about an equal number of males and females. Average family income was slightly less than 3 million Korean won (approximately, $3000 USD). About 9% of the sample had parents who completed less than high school, 45% had completed high school, and the remaining 45% had some college education or more. The majority of youth lived with both parents, with less than 7% of them living with only a father, a mother, or neither parent. Teacher attachment was at low to moderate levels (*M* = 2.45, *SD* = 0.82). Average educational aspiration was between junior college and 4-year college (*M* = 3.89, *SD* = 0.89). About a quarter of the sample youth participated in school extracurricular activities. Finally, rule internalization was relatively high (*M* = 4.14, *SD* = 1.03).

**Table 1:** Descriptive statistics at baseline.

Variables	*M (%)*	*SD*	*Range*	*n*
*School Factors*				
Teacher attachment	2.46	0.82	1–5	3449
Educational aspiration	3.89	0.89	0–5	3449
Extracurricular activity	0.24	0.43	0–1	3449
Rule internalization	4.14	1.03	1–5	3415
*Youth Demographics*				
Male	50%			3449
*Family Characteristics*				
Monthly family income (1,000,000 Korean won)	2.99	2.14	0.05–35	3434
Parental education				
Less than high school	9%			3413
High school	45%			3413
At least junior college	45%			3413
Family structure				
Lives with both parents	94%			3444
Lives with father only	2%			3444
Lives with mother only	3%			3444
Lives with neither	1%			3444

Pearson correlations among the four school bond elements were low ranging from 0.001 (correlation between rule internalization and club activities) to 0.166 (correlation between rule internalization and teacher attachment). These results suggest that the elements of school bonds in this study are distinct to each other and are not subject to multicollinearity.

**Table 2:** Pearson correlations among school bond elements at baseline.

School Bond Elements	(1)	(2)	(3)	(4)
(1) Teacher attachment	1			
(2) Educational aspiration	0.117	1		
(3) Club activity	0.037	0.064	1	
(4) Rule internalization	0.166	0.129	0.001	1

Prior to the first wave of the panel study (up to age 14), 528 and 190 youth experienced alcohol drinking and cigarette smoking for the first time, respectively. By the end of age 19, approximately 71% and 28% of youth experienced alcohol drinking and cigarette smoking, respectively.

**Table 3:** Frequency and cumulative percentage of youth with experience of first-time substance use (N = 3449).

Age	Alcohol Drinking		Cigarette Smoking	
	Frequency	Cumulative (%)	Frequency	Cumulative (%)
6	5	0.14	0	0
7	7	0.35	2	0.06
8	4	0.46	2	0.12
9	4	0.58	3	0.2
10	15	1.01	5	0.35
11	16	1.48	5	0.49
12	72	3.57	23	1.16
13	194	9.19	70	3.19
14	211	15.31	80	5.51
15	534	30.79	275	13.48
16	340	40.65	133	17.34
17	416	52.71	140	21.4
18	322	62.05	130	25.17
19	321	71.35	101	28.1
No experience up to 19	988	-	2480	-

Note: Adopted from Table 2 of Han and Grogan-Kaylor [40].

### 3.2. Survival Analysis: Discrete-Time Logistic Regression

Logistic regression coefficients of the discrete-time hazard model were estimated to predict the likelihood of first-time initiation of alcohol and cigarette use (Table 4). Coefficients represented the conditional probability of engaging in substance use given that the event had not yet occurred [48]. As shown in results of Models 1 and 3, there was no specific upward or downward pattern in the baseline hazard for alcohol drinking and cigarette smoking, respectively. Findings from the alcohol model (Model 4) are as follows. Stronger attachment to teachers, higher educational aspirations, and higher rule internalization scores were statistically significantly associated with delayed onset of youth alcohol use. However, youth engagement in school extracurricular activities was associated with later first-time alcohol use. Males were more likely to experience earlier alcohol use. A higher level of parental education was associated with delayed first alcohol use, whereas income was not related to onset of alcohol use. In terms of family structure, youth living with a father only were more likely to experience alcohol consumption at an early age, compare to youth living with both parents.

Results from the cigarette model (Model 3) are as follows. As for the school bond variables, teacher attachment was associated with delayed first-time smoking. Educational aspiration, an indicator of a youth’s commitment, was associated with delayed first-time smoking. School extracurricular activities, which represent social involvement, did not have a statistically significant relationship with the onset of cigarette use. Rule internalization, which represents the degree to which youth accept the values and beliefs of society, was associated with delayed initiation of cigarette use. Male youth, compared to female youth, predicted earlier initiation of smoking. Family socioeconomic measures, including income and parental education, were not significantly associated with onset of cigarette use. Family structure, on the other hand, showed some statistically significant patterns. Youth who live with a mother only or have no parents experienced early initiation of smoking compared to youth from two-parent families.

**Table 4:** Discrete time logistic regression: onset of alcohol drinking (n = 9242) and cigarette smoking (N = 12,972).

Variables	Alcohol Drinking		Cigarette Smoking	
	Model 1	Model 2	Model 3	Model 4
*Duration ^a^*				
Age 16	−0.143^†^	−0.052	−0.599 ***	−0.508 ***
	(0.078)	(0.079)	(0.113)	(0.116)
Age 17	0.332 ***	0.383 ***	−0.435 ***	−0.348 **
	(0.075)	(0.076)	(0.111)	(0.115)
Age 18	0.298 ***	0.368 ***	−0.510 ***	−0.434 ***
	(0.082)	(0.083)	(0.115)	(0.118)
Age 19	0.660 ***	0.818 ***	−0.598 ***	−0.410 **
	(0.084)	(0.086)	(0.124)	(0.127)
*School elements*				
Teacher attachment		−0.198 ***		−0.130 **
		(0.034)		(0.050)
Educational aspiration		−0.125 ***		−0.275 ***
		(0.032)		(0.041)
Club activity		0.272 ***		0.029
		(0.061)		(0.090)
Rule internalization		−0.208 ***		−0.415 ***
		(0.028)		(0.036)
*Controls*				
Male		0.119 *		0.674 ***
		(0.055)		(0.080)
Monthly family income		0.009		0.022
		(0.015)		(0.020)
Parent’s education		−0.068 **		−0.011
		(0.023)		(0.033)
Lives with father only ^b^		0.485 *		0.313
		(0.193)		(0.246)
Lives with mother only ^b^		−0.119		0.393 *
		(0.143)		(0.184)
No parents ^b^		0.043		0.856 *
		(0.507)		(0.414)
Constant	−1.544 ***	0.427 *	−2.412 ***	0.147
	(0.049)	(0.201)	(0.064)	(0.254)

Notes: Standard errors in parentheses; *** *p* < 0.001; ** *p* < 0.01; * *p* < 0.05; ^†^ *p* < 0.1; ^a^ Reference group is Age 15; ^b^ Reference group is “Lives with both father and mother.” Sample size in survival models are naturally inflated as data from multiple waves are considered as individual points of observation in the analysis. Total of 2870 youth constitute the N of 9242 in the discrete time logistic regression models for alcohol drinking. Total of 3210 youth constitute the *N* of 12,972 in the discrete time logistic regression models for cigarette smoking.

## 4. Discussion

This study explored the association between elements of social bonds within the school and the onset of alcohol and cigarette use during adolescence among Korean youth. While some studies in the past have tested the link between social bonds and youth risk behaviors, to our knowledge, the current study is the first to use the social control theory framework to specifically examine the relationship between four elements of school bonds and first-time initiation of substance use among a nationally representative Korean youth population. In this study, school factors that represent the four elements of Hirschi’s social bond—attachment, commitment, involvement, and belief—were teacher attachment, educational aspiration, extracurricular activity, and rule internalization, respectively. In accordance with the premises suggested by social control theory, we found that teacher attachment, educational aspiration, and rule internalization were significantly associated with delayed first-time substance use. However, school extracurricular activity was inversely associated with first-time drinking and not statistically significantly associated with first-time smoking. A detailed discussion of the results for each of the social bond elements are provided below.

### 4.1. Attachment/Teacher Attachment

Youth with higher teacher attachment significantly showed a delayed onset of alcohol use. A higher level of teacher attachment was also associated with delayed first-time cigarette use at the level of a trend (*p* < 0.10). These results suggest that students who form strong social bonds with their teachers are more likely to start drinking and smoking at a later age than those who care less about their teachers. Our analytic findings support the general idea that positive relationships with teachers encourage positive social and developmental outcomes and prevent antisocial outcomes among youth [18]. More specifically, these results were in line with prior research that found a negative association between teacher attachment and delinquency [1,20,26,55]. In the school setting, school norms or rules are mostly enforced by the teacher as a school authority over students. When teachers provide feelings of support, understanding, respect, and appreciation in the classroom, adolescents gain more interest and enjoyment from their bonds to school and teachers [18], and thus are more likely to comply with teachers’ expectations. Such positive interpersonal relationships can promote youth engagement in school and prevent youth from exhibiting risk behavior [18,55].

### 4.2. Commitment/Educational Aspiration

As predicted, discrete time logistic analysis models reported that educational aspiration was correlated with a delayed onset of students’ alcohol and cigarette use. Consistent with existing research, the findings of this study provide support for the idea that adolescents who are committed to meeting the goals set by conventional society are less likely to be distracted or engage in risk behaviors. For example, in a study among at-risk high school youth attending dropout prevention/recovery high schools, Grunbaum and colleagues [28] reported that students who considered high school to be their highest educational plans were more than two times more likely to use cocaine than those with higher educational aspirations. Aligned with Hirschi’s theory, our results confirmed the vital role of educational ambition and aspiration in developing youth’s commitment toward the conventional school system, while constraining youth from engaging in risk behaviors. Put another way, adolescents who are greatly vested in meeting educational goals concurrently anticipate the high costs of losing their investments by deviating from those goals. Thus, these youth may have rationally decided to delay their onset of alcohol or cigarette consumption.

### 4.3. Involvement/Extracurricular Activities

In the current study, school extracurricular activities were linked with earlier first-time alcohol use, but its relationship with first-time cigarette use was not statistically significantly associated. These findings were inconsistent with our hypothesized negative relationship between school extracurricular activities and substance use. A closer examination of various pathways through which school extracurricular activities influence adolescent risk behavior may help explain these inconsistent study results. Feldman and Matjasko summarized two pathways of influence to explain the mixed results of the effect of extracurricular activities on youth outcomes in the literature [29]. In accordance with the social control theory perspective, extracurricular activities inhibit youth substance use by promoting positive values and behaviors. It is possible, however, that extracurricular activities may increase the likelihood of substance use by greater exposure to peers who engage in risk behavior. Furthermore, literature points to various factors, such as type of activity (e.g., athletics, arts, music, academics), number of activities, the level of participation, or gendered effects, that may exert different influences on a youth’s motivation to engage in risk behaviors [21,29]. For example, Elder and his colleagues found youth with moderate levels of participation in school activities were correlated with less substance use than those with no participation or high levels of participation [31]. Furthermore, Crosnoe found different results corresponding to the types of activity and sex [30]. According to his findings, male athletes and non-athletes showed increased use of substances over time. Females followed a similar pattern, but displayed a lower rate of over-time increase in substance use. The type of friendships that are developed through extracurricular activities may also attribute to the positive relationship between extracurricular activities and adverse youth outcomes, as there is evidence that peer relationships are associated with increased probability of engaging in various health-risk behaviors [26]. The current study, which utilized information from a secondary dataset, was not able to take into consideration such intervening factors. Future work should consider more detailed aspects (e.g., specific type, number of activities, and time spent) and the nature of peer relationships that are formed through school activities.

### 4.4. Belief/Rule Internalization

Of the four social bond elements, the construct of belief has been operationalized in multiple ways, including attitudes toward risk behavior [34], legitimization of the legal system [1], importance or relevance of social norms in youth’s lives [21], and evaluation of school rules [35]. The current study measured belief as a concept representing internalization of school norms by asking whether school rules and regulations are difficult to follow. Analytic findings indicated that youth who have greater difficulty abiding by the school norms are likely to experience early onset of alcohol drinking and cigarette smoking behaviors. Study findings are consistent with literature, which has documented the negative link between youth’s belief in the moral values and norms of a social system and risk behavior in various sociocultural contexts [35,36]. Internalization of the common value system that is accepted in society has been found to be a protective factor that is associated with delayed onset of substance use behaviors among youth.

### 4.5. Other Covariates

Although Hirschi focused on the role of social bonds in predicting youth substance use behaviors, findings related to family variables analyzed in the current study may warrant further discussion, particularly since some results seem contradictory. For example, parent’s education was only significant for delayed onset of alcohol drinking, but not cigarette smoking. Furthermore, household income was not a significant predictor of onset of youth substance use. Although there seems to be a weak general consensus on the nature of the association between socioeconomic status and adolescence smoking [56] and alcohol [50], results of the current study replicate prior work that have found that education is more consistently related to substance use than household income [49]. In terms of family structure, youth from families with father only predicted earlier onset of alcohol drinking, compared to youth living with both parents. For cigarette drinking, however, youth residing with mother only or not living with any parents were associated with early onset, compared to living with both parents. These results were consistent with the literature that postulates the robust protective role of two-parent families [51]. Further investigation of the family processes that lead to different results in mother-only of father-only families for the alcohol and cigarette models is warranted in future research.

### 4.6. Limitations and Suggestions for Future Work

Several limitations should be considered when interpreting the findings of the current study. First, youth who had already experienced their first risk behavior prior to wave 1 and were omitted from the discrete-time logistic regression portion of the analysis (a.k.a., left-censored youth) may bias analytic results. Post-hoc auxiliary analyses confirmed that the analytic and the omitted youth samples were statistically different (*p* < 0.05) in terms of baseline characteristics. For example, compared to the analytic youth, left-censored youth in the alcohol initiation model were more likely to be female with lower teacher attachment and school adjustment, but more likely to participate in club activities. In the cigarette initiation model, however, there was no difference in individual demographic characteristics at baseline. Instead, omitted youth were more likely from father-only families with lower parental education and reported lower levels of all four elements of school bonds. These results suggest that youth with specific demographic and family characteristics, and relatively low school bonds were likely to have been omitted (or left-censored) from the event history analysis portion of the study. This selection issue may possibly lead to biased estimates of the coefficients on predictors concerning the four elements of school bonds and may limit interpretation of the study findings.

Second, although the current study utilized longitudinal data, results do not guarantee causal interpretation. Prior work has confirmed the possibility of bidirectional effects in which social bond influence youth risk behaviors, but risk behaviors also influence the extent to which youth form social bonds over time [55]. In other words, although youth with strong social bonds are less likely to engage in risk behaviors, it is also equally possible that delinquent behaviors cause lower attachment to teachers, lower educational aspirations, fewer participation in school extracurricular activities, and limit internalization of school norms. Thus, a detailed investigation of the exact temporal processes is warranted in future research to clarify causal relationships between social bonds and youth risk behaviors.

Third, one important covariate that was not considered in the present study is parental use of substances. Prior research using longitudinal data have reported the influential role of parent’s current and past substance use behaviors in predicting substance use behaviors of their offspring. For example, previous studies have pointed to the fact that parental use of substances elevates the risk of youth’s alcohol use [57,58], problematic alcohol use [59], cigarette use [60], and smoking initiation [61]. However, due to data unavailability in using secondary data, this information was not accounted for in the model. Future work should closely investigate the intergenerational influences of substance use on youth substance use behaviors. 

Finally, the current study treated and tested the four elements of social bonds, with the assumption that they operate distinctly, to explain an individual’s decision to engage in risk behaviors. However, these four elements can also be interdependent [1]. For example, youth who are attached to teachers (“attachment”) may be more likely to commit to educational goals (“commitment”), engage in conventional activities (“involvement”), or accept and abide by school norms (“belief”). It is equally plausible that highly committed youth are more involved in school activities and make greater effort to develop strong bonds with teachers. Literature pertaining to social control theory and youth risk behaviors would benefit by testing the causal relationship across the four bond elements and further examining the extent to which the four elements are connected using longitudinal information.

## 5. Conclusions

Notwithstanding the aforementioned limitations, this study contributes to the empirical and theoretical literature concerning substance use among youth with a sample that is currently understudied. Particularly, based on the social control theoretical framework, the current study provides a comprehensive examination of the relationship between the four elements of Hirschi’s social bond and the early onset of risk behavior using a nationally representative data of Korean youth. Results from discrete-time logistic regression generally supported the predictions of social control theory such that school-related factors that represent social bond were associated with delayed first-time use of substances during adolescence. In detail, teacher attachment, educational aspiration, and rule internalization were protective factors for onset of youth alcohol and cigarette use. School club activity, however, was linked with an earlier first-time alcohol use while it was not a significant predictor of first-time cigarette use. These findings suggest that early prevention strategies for youth substance use should specifically target school-related factors that represent social bonds that youth develop. For instance, to increase youth attachment to teachers, teachers should be encouraged to become role models to their students and provide support and respect in the classrooms. For higher educational aspiration and rule internalization, school officials and teachers should promote the value and importance of educational goals and social norms among youth. Finally, more attention should be paid to school extracurricular activities and its influence on youth risk behavior. Schools should examine the different goals and characteristics of extracurricular activities as well as regularly monitor students’ activities so that participation would not inadvertently lead to youth exposure to negative peer influence.
